# Modifications in fire frequency impact belowground plant components in old‐growth grasslands, posing risks to their resilience

**DOI:** 10.1002/ajb2.70108

**Published:** 2025-10-10

**Authors:** Aline Bertolosi Bombo, Alessandra Fidelis, Soizig Le Stradic

**Affiliations:** ^1^ Lab of Vegetation Ecology, Instituto de Biociências Universidade Estadual Paulista (UNESP) Avenida 24‐A, 1515 Rio Claro 13506‐900 Brazil; ^2^ UMR Biogeco, Université Bordeaux, INRAE Allée Geoffroy Saint‐Hilaire, Bâtiment B2 ‐ CS 50023 Pessac 33615 Cedex France

**Keywords:** belowground traits, bud bank, campos rupestres, edaphic grasslands, fine roots, nutrient‐poor soils, resprouting organs, rhizomes, root biomass, storage organs

## Abstract

**Premise:**

In edaphic old‐growth grasslands, the low nutrient content of the soil restricts plant species establishment. Additionally, fire remains an important factor in shaping vegetation structure and belowground biomass investment in old‐growth grasslands, such as the campos rupestres. However, how fire frequency affects the belowground components of these grasslands remain poorly understood. Addressing this gap is essential for understanding plant resilience and regeneration strategies in fire‐prone ecosystems worldwide and advancing trait‐based perspectives on plant persistence under recurrent disturbances.

**Methods:**

We compared plant belowground components across three campos rupestres sites differing in fire frequencies (1, 6, and 10 fires in 34 years). At each site, we quantified fine root biomass, specialized belowground organ biomass, bud bank size, bud‐bearing organ density, and organ composition to evaluate how repeated fires shape belowground traits related to persistence and regeneration.

**Results:**

High fire frequencies reduced bud bank size and belowground organ density, but altered organ composition: Graminoid and forb rhizomes declined in number, whereas woody rhizomes became more common. Overall, belowground organ biomass increased with increasing fire frequency, but fine root biomass decreased.

**Conclusions:**

Our study indicates that changes in belowground components driven by fire frequency may lead to irreversible shifts in community structure, where very frequent fires can jeopardize the resilience of the campos rupestres. For such systems, novel fire regimes can have devastating effects, threatening biodiversity, compromising conservation status, and reducing ecosystem services.

Old‐growth grasslands host an immense plant diversity, characterized by high herbaceous species richness, high endemism, and unique species composition (Veldman et al., 2015; Nerlekar and Veldman, 2020). The campos rupestres are tropical old‐growth grasslands constrained mostly by edaphic and climate conditions (Silveira et al., 2016), and that are prone to fire (Veldman et al., 2015; Buisson et al., 2019, 2022). Thus, plants in these ecosystems present traits that allow thriving constraint edaphic conditions as desiccation tolerance (Brum et al., 2017; Teodoro et al., 2021), drought avoidance (Brum et al., 2017), but also recovery ability (Le Stradic et al., 2018c; Araújo and Conceição, 2021). They also may have a large diversity of root types to cope with contrasting nutrient‐impoverished soil types (Oliveira et al., 2015, 2016). Limited water and soil nutrient content greatly impacts belowground traits and nutrient acquisition. Due to contrasting strategies to acquire water, grass and sedge species present a clear functional divergence compared to other species, with fasciculate roots reaching only shallow soils (Díaz et al., 2016; Brum et al., 2017), whereas forbs and woody species can present great variation in rooting depths and belowground specialized organs (Moraes et al., 2016; Brum et al., 2017; Joaquim et al., 2018; Da Silva et al., 2023).

These tropical old‐growth grasslands are not strictly fire‐dependent (Pivello et al., 2021), but several clues have indicated that fire plays a role in structuring them (Figueira et al., 2016). They are highly resilient to fire (Neves and Conceição, 2010; Le Stradic et al., 2018c) since plants have strategies to persist and survive after a fire, including the presence of storage in specialized belowground organs, coupled with the capacity to repeatedly resprout from bud‐bearing organs after aboveground biomass removal (Moraes et al., 2016; Joaquim et al., 2018; Da Silva et al., 2023), and fire‐related cues for germination (Le Stradic et al., 2018a; Zirondi et al., 2019a, 2019b). Fire exclusion can lead to changes in species composition, such as a lowered species richness (Le Stradic et al., 2018c), indicating that these grasslands are maintained through interactions between fire and soil characteristics (Buisson et al., 2019).

The most common post‐fire regeneration strategy in these systems is resprouting (Neves and Conceição, 2010; Figueira et al., 2016; Le Stradic et al., 2018b), which is enabled by the presence of viable bud banks. Bud banks can be aerial, where buds are protected either by the presence of a thick bark (Simon and Pennington, 2012; Chiminazzo et al., 2023) or overlapped leaves (aboveground buds, Lusa et al., 2014) or by the soil when located on belowground specialized organs (Pausas et al., 2018). Additionally, belowground organs must supply resources for resprouting, either by producing roots to uptake soil nutrients (Da Silva et al., 2023) or by investing in storage organs (Moraes et al., 2016; Klimešová et al., 2018; Pausas et al., 2018). In fire‐prone systems, belowground bud bank maintenance is triggered by frequent fires (Fidelis et al., 2014; Bombo et al., 2022a, 2022b). However, this ability may be hampered by different fire regimes, particularly alterations in fire frequency (Vesk and Westoby, 2004; Figueira et al., 2016), which can affect the density of viable buds and the composition of the belowground bud‐bearing organs (Fidelis et al., 2014; Bombo et al., 2022a, 2022b).

Therefore, novel fire regimes can significantly affect not only the responses from the aboveground plant community (Le Stradic et al., 2018c), but also the belowground components of the vegetation, affecting how plants will regenerate after a disturbance. In tropical open savannas, recurrent fires are important to maintain belowground bud‐bearing organ composition, bud banks, and fine roots. Fire exclusion, however, leads to a decline in bud‐bearing diversity, favoring the dominance of fleshy rhizomes from a few grass species (Bombo et al., 2022a). This process can result in the disappearance of large structures like xylopodium, basal woody burls that store a large number of belowground renewal buds (Pausas et al., 2018), ultimately compromising the system's resilience (Bombo et al., 2022a). Roots also respond quickly to changes in fire frequency because studies have shown an increase in fine root biomass following frequent and recent fires (Oliveras et al., 2012; Le Stradic et al., 2021), in response to the demand for resources needed to promote shoot regrowth. However, in nutrient‐poor ecosystems such as campos rupestres, where nutrients are scarce, plants may be limited in their ability to produce large amounts of roots after each fire, potentially hindering their capacity to thrive under frequent fires.

Although plants from campos rupestres also rely mostly on belowground storage organs and bud banks for post‐fire resprouting (Silveira et al., 2016; Le Stradic et al., 2018b), they may respond differently than other old‐growth grasslands (e.g., savannas) to different fire regimes, since it is also edaphically constrained. Campos rupestres have a moisture‐dependent fire regime, with lower fire frequencies (every 6–8 years, Figueira et al., 2016; Alvarado et al., 2017) than tropical savannas (every 1–5 years, Eiten, 1982). Changes in fire regimes in campos rupestres are expected due to anthropogenic actions (i.e., human‐induced fires) or due to new climatic conditions and might affect plant post‐fire responses. In general, fire‐prone systems may experience increased fire frequency, fire season length, and risks of more severe fires because of significant decreases in precipitation (Senande‐Rivera et al., 2022). Thus, it is crucial to understand how novel fire regimes (e.g., lower or higher fire frequencies) would affect the belowground components, since they will be responsible for providing resilience to the vegetation, even in edaphic‐constrained grasslands.

Therefore, we investigated how belowground traits respond to different fire frequencies in campos rupestres. We analyzed the belowground bud bank density, bud‐bearing belowground organs (BBOs) composition and biomass, root biomass, and BBOs‐to‐shoot and root‐to‐shoot biomass ratios in sites with different fire frequencies: low, intermediate, and high fire frequencies. Since the intermediate fire frequency represents the natural fire regime, we hypothesized that the highest accumulation of buds in the bank and fine root biomass, along with the greatest diversity of belowground organs, would be found in areas with this fire frequency. If fire frequency increases (high), then we expect a loss of buds in the bud bank resulting from biomass removal and successive resprouting and a decrease in fine roots caused by the burning of shallow roots and reduced root production due to repeated burning. Coupled with low edaphic resources, these combined effects may strongly limit the ability to refill the bud bank and produce new roots. Therefore, bud‐bearing organs would perish in the system. On the other hand, if fire is excluded for longer periods (low), then more aboveground biomass will be allocated to aboveground compartments, and the composition of belowground organs will significantly change. In such conditions, grasses will outcompete forb species by excluding them through biomass accumulation.

## MATERIALS AND METHODS

### Study site

The study was located in the Parque Nacional Serra do Cipó and its buffer zone (Morro da Pedreira Environmental Protection Area) in the southern portion of the Espinhaço mountain range, in southeastern Brazil (19°17′ S, 43°33′ W, 700–1670 m a.s.l.; Appendix S1: Figure S1). The climate in the area is warm tropical and seasonal with marked cold dry winters and hot wet summers (Silveira et al., 2016). The annual mean rainfall is ca. 1400 mm, most of it during the rainy season (October–April; Alvarado et al., 2017). The mean daily maximum and minimum temperature is, respectively, 33°C and 28°C at the peak of the hot wet summer and 13°C and 7°C at the peak of the dry cold season (Alvarado et al., 2017).

The vegetation of the region is primarily campos rupestres, a mosaic of old‐growth grasslands at high altitudes, composed of a species‐rich herbaceous layer with scattered woody species occurring mainly in the rocky outcrops. They have a high level of endemism, and the main families are Poaceae, Cyperaceae, Eriocaulaceae, Asteraceae, and Xyridaceae (Giulietti et al., 1987; Silveira et al., 2016). The vegetation types composing campos rupestres include wet grasslands, stony grasslands, or sandy grasslands. Campos rupestres are snow‐free despite occurring at altitudes higher than 900 m on shallow and sandy soils over quartzitic bedrock (Giulietti et al., 1987; Silveira et al., 2016). Campos rupestres are also fire‐prone environments with a fire return time of 6 to 8 years in the Parque Nacional Serra do Cipó, mainly occurring during the dry season (Figueira et al., 2016; Alvarado et al., 2017).

We selected three sampling sites in sandy grasslands, presenting different fire frequencies: 1 to 10 fires from 1984 to 2018 (i.e., a proxy of fire frequency, Appendix S1: Table S1, Figure S1). In the region, 55–49% of all burned areas was burned between one and four times from 1984 to 2015, 33–20% was burned between five and nine times, and only 2–3% was burned 10 or more times during the same period (Alvarado et al., 2018). Using these three categories, we selected sites with low (one fire event in 35 years), intermediate (six fire events in 35 years), and high (10 fire events in 35 years) fire frequencies. Each site had a different length of time since the last fire, ranging from four years (intermediate and high) to 19 years of fire exclusion (Appendix S1: Table S1).

### Biomass sampling

In each site, we sampled belowground organ and root biomass and the associated aboveground biomass. To assess the aboveground biomass and belowground bud‐bearing organ biomass and bud bank size at each of the three sampling sites, we randomly established ten 0.5 × 0.5 m plots, maintaining a minimum distance of 20 m between plot edges. Within each bud bank plot, aboveground biomass was entirely removed 5 mm above the ground and bagged separately. At the laboratory, standing dead and live parts were separated, oven‐dried at 80°C for 48 h, then weighed. To assess belowground organ biomass, we excavated the plots down to 10 cm and directly sieved the soil to remove part of the soil and large stones. Since the majority of belowground resprouting buds are located between 0 and 5 cm deep (Klimešová et al., 2019), samples were collected only from depths of 0–10 cm. All belowground plant structures were collected in a plastic bag and stored in a cooler for transport for further analysis at the laboratory, including bud counting. The sampling was performed during the rainy season (April 2018) to account for dormant buds that accumulated during the previous growing season, representing the maximum productivity of the studied plant communities (Klimešová et al., 2019). At the laboratory, belowground structures were water‐washed to remove most of the soil attached to roots and other organs. Roots attached to the different organs, primarily adventitious roots in grass rhizomes, were removed to assess only belowground bud‐bearing organ biomass. All other plant parts, including belowground bud‐bearing organs and those that could be ambiguous in their potential to bear buds, were retained for further detailed investigation. All the belowground structures were stored in 70% ethanol for later bud bank analyses.

For the bud bank analysis, live buds from all perennial organs found in each 0.25‐m² sampled plot were counted. The number of buds for each plot was summed, and the mean and standard error were calculated for each site (treatment). The bud bank size was then extrapolated to estimate the number of buds per square meter. Belowground buds were also counted according to growth forms and categorized as graminoids or non‐graminoids (including forbs sensu Siebert et al. [2024]) and shrubs. The bud‐bearing structures were classified into specific belowground organ types according to morphological types as described by Pausas et al. (2018). Among the belowground organ types, we recognized the following categories, adapted from Pausas et al. (2018): non‐graminoid rhizomes, comprising all types of fleshy rhizomes from forbs; graminoid rhizomes, composed of fibrous rhizomes from grasses and sedges; bulbs, including all swollen structures with one to a few buds, which may also include corms and stem tubers; root crowns, bearing a substantial number of buds at the root collar; xylopodia, encompassing all woody basal burls, regardless of their developmental origin; woody rhizomes, including true woody stems (*soboles*) that grow horizontally under the soil surface and woody gemmiferous roots, which can only be distinguished from *soboles* through detailed anatomical analysis; and, other, which includes fragmented structures that could not be accurately classified and were considered in bud bank analyses only if they bore live buds (Bombo et al., 2022a). Buds were counted using a stereomicroscope. After bud counting, all the belowground plant material was transferred to paper bags, oven‐dried at 80°C for 48 h, and weighed. The belowground organ‐to‐shoot (BBO:shoot) ratio was calculated as the ratio of belowground bud‐bearing organ biomass in the first 10 cm of soil to total live dry aboveground biomass (collected in the respective plots; g·m^–2^).

For assessing root biomass, we randomly set up five circular plots of 0.25 m^2^ in each sampling site in February 2018. In each root plot, total aboveground biomass was sampled as described previously. As previously mentioned, aboveground biomass was separated into dead and live fractions, oven‐dried at 80°C for 48 h, and only the live fraction was weighed to determine the live aboveground dry biomass per square meter. In the middle of each plot, we collected soil samples from the soil surface down to a 20‐cm depth using an auger (5 cm diameter). Harvested roots represented a mixture of roots from species occurring above and in the vicinity of the core. Samples were stored at –18°C before analysis. Soil cores were defrosted at the laboratory and gently washed with water using 1 mm, 850 µm, and 200 µm sieves to avoid losing roots (Pérez‐Harguindeguy et al., 2013). Fine roots (<2 mm) were then separated, and each root sample was oven‐dried at 60°C for 72 h (Pérez‐Harguindeguy et al., 2013) and weighed. The root‐to‐shoot ratio (root:shoot) was calculated as the ratio of fine root biomass (i.e., roots <2 mm) in the first 20 cm of soil to live aboveground biomass (collected in the respective plots; g·m^−2^).

### Data analyses

We tested whether the bud bank density and related belowground traits varied among sites with the different number of fires (1, 6, and 10 fires between 1984 and 2018; hereafter referred to as fire frequency). Specifically, we evaluated differences in bud bank density (number of buds·m⁻^2^, both total and by growth form), proportion of live belowground bud‐bearing organs (% of the total for each belowground organ type), the density of each belowground organ type (number of belowground organs·m^–2^), belowground organ‐to‐shoot ratio, and the root‐to‐shoot ratio.

Generalized linear mixed models (GLMMs) were used for these analyses The models were fitted using the glmmTMB package (Brooks et al., 2017), with response variables modelled as a function of fire frequency, while plots were treated as random factors when applicable (Zuur et al., 2009). Different distributions, such as Poisson and negative binomial, were tested to address potential overdispersion, and the Poisson family with a logit link function was applied for count data. Zero‐inflated models were applied for variables with excess zeros, using the zeroinfl function in the R package pscl (Jackman, 2020). For all tested variables, multiple pairwise comparisons were conducted using Tukey's honest significant difference (HSD) test to assess differences between treatments. Model selection was performed using likelihood ratio tests and information criteria, such as the Akaike information criterion (AIC) and Bayesian information criterion (BIC), to identify the most parsimonious model. Residuals were assessed using the DHARMa package (Hartig, 2020), and model performance was evaluated with the R package performance and check_model() function (Lüdecke et al., 2021). Type III ANOVA, conducted using the R package car (Fox and Weisberg, 2019), tested the significance of the fixed effects, while pseudo‐*R*² values were calculated to assess model fit. Final models were selected based on the lowest AIC/BIC values, residual diagnostics, and data fit.

To assess whether the proportional allocation of different belowground storage organs differed among fire frequency treatments, we performed permutational multivariate analysis of variance (PERMANOVA) implemented with the function adonis in the R package vegan (Oksanen et al., 2025). The analysis was based on a Bray–Curtis dissimilarity matrix calculated from organ‐type proportions, with 9999 permutations. To identify specific differences between treatments, pairwise comparisons were performed using the function pairwise.adonis.

All statistical analyses were conducted using R version 4.1.2 (R Core Team, 2019). Results were visualized using R packages sjPlot (Lüdecke, 2024) and report (Makowski et al., 2023), and R packages Tidyverse (Wickham et al., 2019) and ggplot2 (Wickham, 2016) for data manipulation and graphical presentation. CorelDRAW X6 version 16.0.0.707 (2012) was also used for visual representations.

## RESULTS

### Bud bank size, belowground organ density, and composition

**Figure 1:**
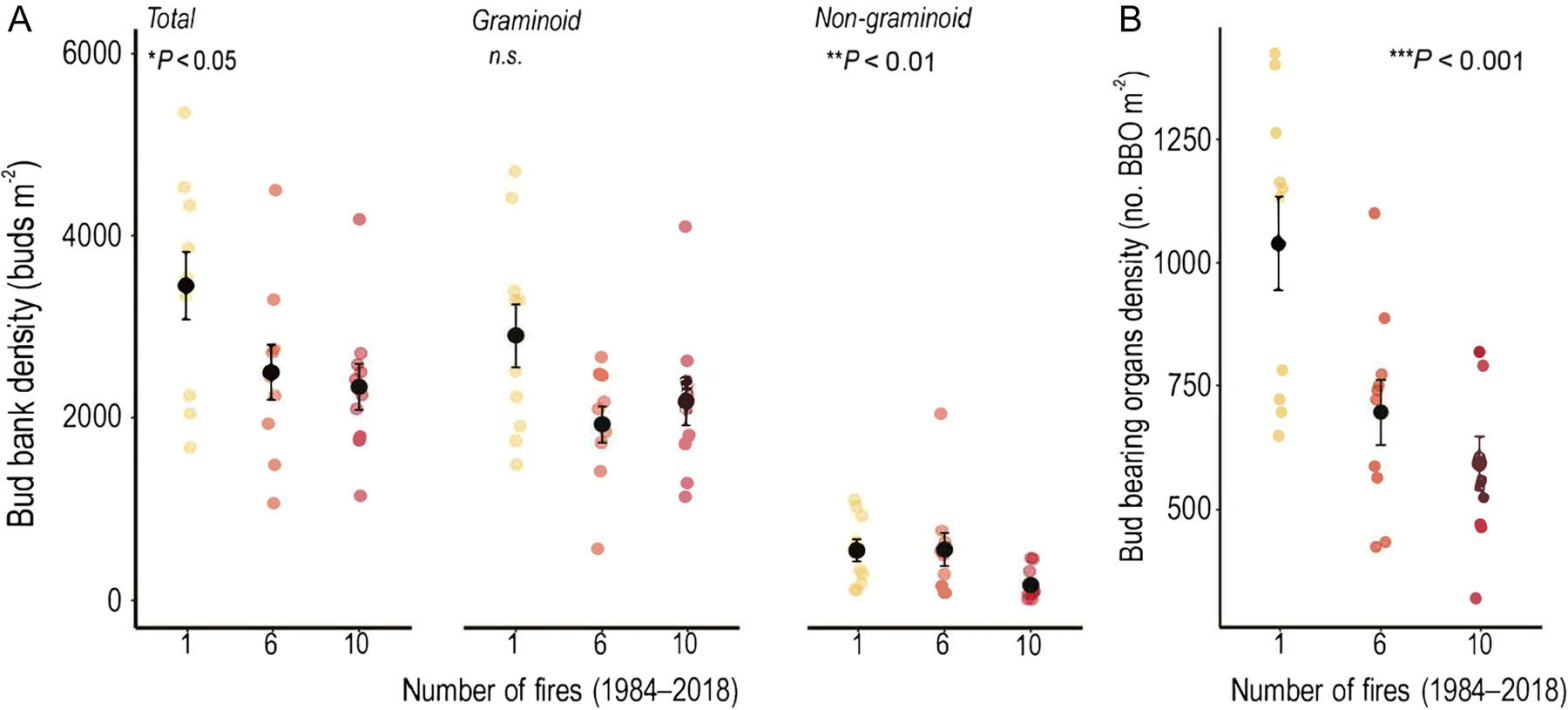
Density of belowground bud bank and belowground bud‐bearing organs at different fire frequencies (1, 6, and 10 fire events from 1984 to 2018) in the campos rupestres. (A) Total belowground bud bank density and from graminoid (grasses and sedges) and non‐graminoid species (forbs and shrubs). (B) Density of belowground bud‐bearing organs. Colored dots represent data from each plot; black dots represent means; bars indicate standard error. For more details, see Appendix S1: Table S2.

The site with low fire frequency (1‐fire site) had a higher total bud bank (*P* < 0.05, Figure 1A; Appendix S1: Table S2), with 47% higher density compared to the high fire frequency site (10‐fire events), and 38% more buds compared to the intermediate fire frequency site (6‐fire events). Similar bud bank values were found between intermediate and high fire frequency sites (6‐ and 10‐fire events, respectively). The graminoid bud bank was the major component of the total bud bank across all sites with different fire frequencies. For non‐graminoid species, bud density decreased drastically at the high fire frequency site compared to the other sites. Low and intermediate fire frequency sites had similar numbers of buds (Figure 1, Appendix S1: Table S2).

**Figure 2:**
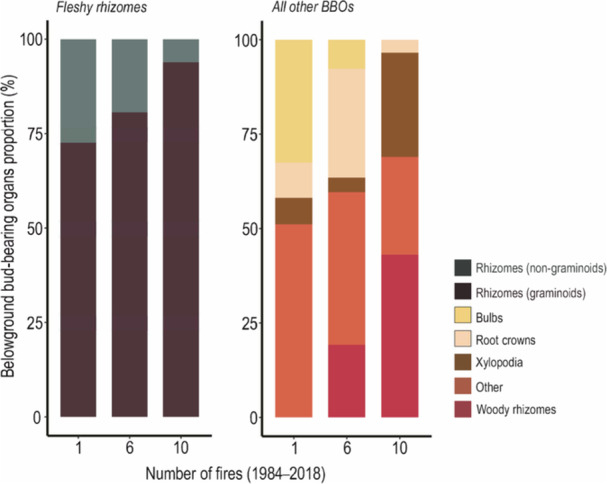
Composition of belowground bud‐bearing organs at different fire frequencies (1, 6, and 10 fire events from 1984 to 2018) in the campos rupestres. Left, fleshy rhizomes; right, all other belowground bud‐bearing organs (BBOs), accounting for approximately 1.7%, 3.0%, and 3.7% of the total in 1‐fire, 6‐fire, and 10‐fire sites, respectively. For more details and statistics, see Appendix S1: Tables S2 and S3.

A greater accumulation of belowground organs was found in the 1‐fire site compared to intermediate and high fire frequencies (*P* < 0.001, Figure 1B; Appendix S1: Table S2). We found that the proportions of belowground organ types differed significantly among the sites (*R* ^2^ = 0.31, *F* = 12.3, *P* = 0.0011). Pairwise comparisons showed that sites with 10 fires differed significantly from both the 6‐fire (*P* = 0.021) and 1‐fire (*P* = 0.003) sites, while no significant difference was found between the 6‐fire and 1‐fire sites (Appendix S1: Table S3). All organ types were represented in the 6‐fire site. Woody rhizomes were not found in the 1‐fire site, and bulbs were not found in the 10‐fire site (Figure 2; Appendix S1: Table S2). Fleshy rhizomes represented the majority of belowground organs, especially graminoid rhizomes (Figure 2; Appendix S1: Table S2) in all sites. Non‐graminoid fleshy rhizomes were significantly lower in number and proportion in the 10‐fire site (Figure 2; Appendix S1: Table S2). All other organ types had lower representation in the total number of organs, accounting for approximately 1.7%, 3.0%, and 3.7% in sites with low, intermediate, and high fire frequency, respectively. Fire frequency only significantly affected the total number of woody rhizomes (Appendix S1: Table S2).

### Belowground biomass

Belowground organ and root biomass differed distinctly in response to fire frequency. The biomass of belowground organs did not vary among the three sites, while the biomass of fine roots was 64% higher at the 1‐fire site and 22% lower at the 10‐fire site compared to the intermediate fire frequency site (*P* < 0.001, Table 1).

**Table 1:** Belowground biomass of sampled sites in campos rupestres according to fire frequency (number of fires from 1984 to 2018). Ratio values greater than 1.0 indicate greater allocation to belowground than aboveground biomass. For each linear mixed‐models, we report the incidence rate ratios for the intercept and the treatment, and *P*‐value. The intercept represents the baseline incidence rate, while the treatment effect reflects the relative change in the incidence rate associated with the treatment variable (*P < 0.05, **P < 0.01, ***P < 0.001, n.s. nonsignificant). Different letters represent treatment differences identified by Tukey's HSD test.

No. of fires	Belowground organ biomass (g·m^–2^)	Fine root biomass (g·m^–2^)	Belowground organ:shoot ratio	Root:shoot ratio
1	760.72 ± 52.63 a	621.02 ± 64.21 a	4.9 ± 0.49 a	2.6 ± 0.38 a
6	627.27 ± 103.17 a	378.34 ± 69.17 b	6.75 ± 0.73 ab	1.63 ± 0.19 b
10	873.55 ± 108.77 a	294.34 ± 71.83 b	8.19 ± 1.03 b	1.35 ± 0.16 b
	Model estimates			
(Intercept)	691.92***	639.77***	4.53***	2.66***
Treatment	10.93 n.s.	–36.80***	0.37**	–0.14***
df	26	10	26	11

*Note*: Belowground organ biomass at soil depth of 0–10 cm; fine root biomass at depth of 0–20 cm.

**Figure 3:**
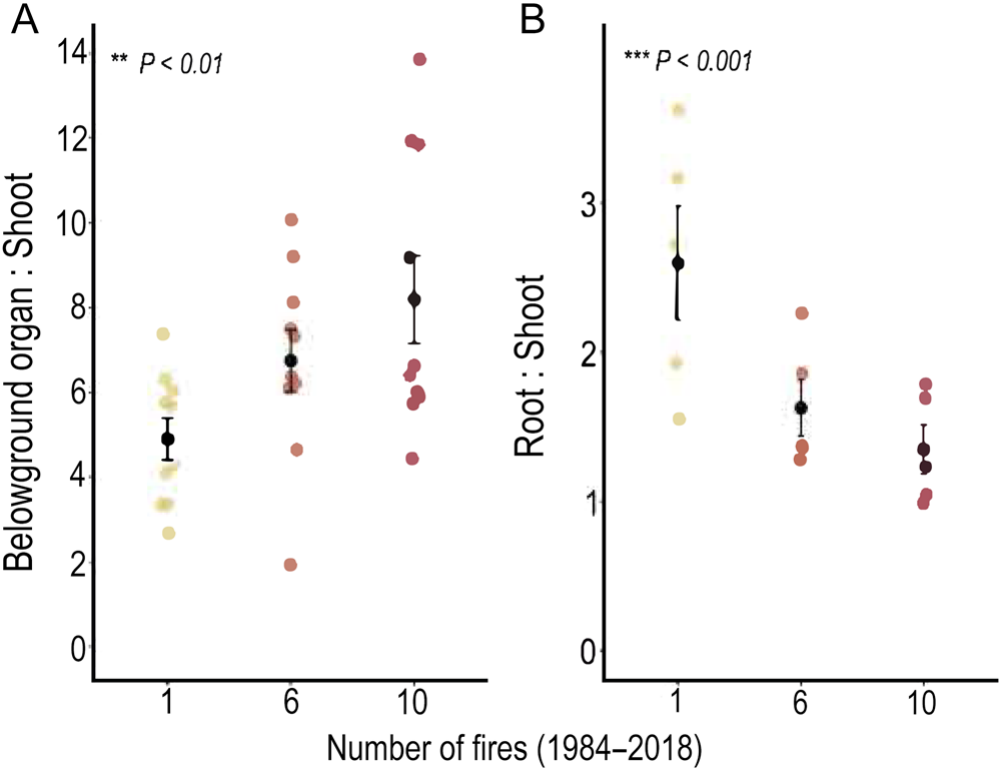
Belowground organ:shoot and root:shoot biomass ratios at different fire frequencies (1, 6, and 10 fire events from 1984 to 2018) in the campos rupestres. Colored dots represent data from each plot; black dots represent means; bars represent standard error. For more details and statistics, see Table 1.

Independent of fire frequency at the sites in the campos rupestres, the belowground biomass (both belowground organs and fine roots) was always higher than aboveground biomass, since the belowground‐to‐aboveground ratio was consistently greater than 1 (Figure 3, Table 1). The belowground organ‐to‐shoot ratio increased with high fire frequency (*P* < 0.01), while the root‐to‐shoot ratio showed the opposite response (*P* < 0.001) (Figure 3, Table 1).

## DISCUSSION

Our study showed that fire frequency is not only an important factor influencing the aboveground plant community but also the belowground components in the campos rupestres. As expected, high fire frequency led to a lower bud bank density and a decrease in the number of belowground organs. In all three sites, fleshy rhizomes of graminoids were dominant, while woody rhizomes increased in relative proportion in frequently burned sites (moderate and high), despite being less important or even absent in site with lower fire frequency. Although belowground organ biomass did not vary across sites, root biomass and both belowground organ‐to‐shoot and root‐to‐shoot ratios did change and were consistently higher than 1.0, indicating greater investment in belowground perennating organs and the resilience of this component to fire. Additionally, the composition of belowground storage organs shifted among the fire frequencies, pointing to a consistent overall investment in belowground organs, irrespective of changes in their composition. Our study supports the premise that changes in fire regimes may compromise the resilience of campos rupestres by altering belowground components, both storage organs and roots. Specifically, an increase in fire frequency negatively impacted bud bank size, with the strongest effects observed in the forb component. Differently from tropical savannas (Bombo et al., 2022a) and subtropical grasslands (Fidelis et al., 2014), in campos rupestres, forbs will profit more under a low and an intermediate fire frequency.

We identified that the total bud bank size decreased under high fire frequency (in our study site, one fire every 3 to 4 years), possibly due to the recurrent removal of aboveground biomass and resprouting, along with insufficient time or resources to replenish the bud bank. Also, storage depletion in belowground organs may contribute to this decline because campos rupestres typically experience fires at an intermediate fire frequency, approximately every 6 to 8 years (Figueira et al., 2016; Alvarado et al., 2017). Additionally, some organ types tend to store more buds than others (Pausas et al., 2018), then bud accumulation may be driven by the number and diversity of belowground organs present (Pausas et al., 2018; Ott et al., 2019; Ferraro et al., 2021; Bombo et al., 2022a). Indeed, at the high fire frequency site, we found that the lower number of buds was mainly due to the lower amount of non‐graminoid bud banks and live belowground organs. The reduction of belowground organs and, consequently, of the total bud bank, is consistent with findings in other grassy systems, where a lower number and diversity of belowground storage organs correspond to smaller bud banks (Fidelis et al., 2014; Ferraro et al., 2021; Bombo et al., 2022a, 2022b). In campos rupestres, the belowground structures of herbaceous species are generally very small, even when they function as storage organs (A. B. Bombo, personal observation). As fire frequency increases, it may become energetically impossible to produce or maintain the necessary storage organs to support new aerial shoots between fires (Bellingham and Sparrow, 2000; Klimešová et al., 2019; Ott et al., 2019), affecting the post‐fire regeneration and, ultimately, the resilience of the system.

The most affected group in terms of bud bank size and number of belowground organs was the non‐graminoid species, which in this study included the forbs and shrubs. In Campos Rupestres, forb species include not only herbaceous and subshrub species (Silveira et al., 2016), but also several monocotyledon families that are characteristic of campos rupestres flora (Giulietti et al., 1987; Silveira et al., 2016). Their representatives possess belowground organ types such as fleshy rhizomes and bulbs (Appendix S1: Table S2). Bulbs, for example, are specialized storage organs of herbaceous plants (Klimešová et al., 2019), commonly associated with the Iridaceae, Alliaceae, and Amaryllidaceae families (Dutilh, 2005). In turn, non‐graminoid fleshy rhizomes are linked to Eriocaulaceae, Xyridaceae, and Velloziaceae families (Giulietti et al., 1987), which are characteristic components of the campos rupestres vegetation (Le Stradic et al., 2015; Silveira et al., 2016). Therefore, unlike the cerrado savannas, where most forbs and shrubs have xylopodia and woody rhizomes, which may dominate the belowground component (Appezzato‐da‐Glória and Cury, 2011; Silva et al., 2021; Bombo et al., 2024; Cozin et al., 2024), bulbs and fleshy rhizomes seem to play a major role in the campos rupestres. Consequently, the reduction of these structures due to increased fire frequency may compromise post‐fire regeneration, especially for characteristic campos rupestres species, leading to changes in the aboveground plant community.

In our samples, monocotyledon families such as Eriocaulaceae, Xyridaceae, and Velloziaceae were very abundant among the forb species (data not shown). The bud‐bearing organs of these families are often located at the soil surface, not benefiting from the soil depth and its insulating properties. However, these structures may exhibit other protective traits for buds positioned at the soil level, such as rosette life forms (Eriocaulaceae, Velloziaceae, Asteraceae, Apiaceae, and others) (Figueira and Del Sarto, 2007; Andrino et al., 2015; Silveira et al., 2016), lignified scales covering the buds (Xyridaceae) (Wanderley, 2010; A. B. Bombo, personal observation), and stems covered by dead, dry leaf sheaths (Velloziaceae) (José and Alves, 1994). Thus, since these structures are more exposed to fire, frequent fires may scorch the viable buds, killing some of them and depleting the bud bank. As we observed, fewer non‐graminoid buds and bud‐bearing belowground organs were produced in the site with high fire frequency (10‐fire) than in sites with intermediate (6‐fire) or low (1‐fire) fire frequency (Appendix S1: Table S2), confirming that forb species were strongly affected by the increase in fire frequency.

One of the key findings of our study is that fire reshapes the composition of the organs in the belowground component, without altering their total biomass. In the campos rupestres, the relative contribution of different belowground storage organs shifted among treatments, suggesting that the system maintains a relatively stable belowground organ load regardless of its composition. However, such compositional changes may have functional consequences because different organ types (e.g., woody rhizomes, bulbs, fleshy rhizomes) vary in their capacity for bud protection, resprouting ability, and resource storage. Similar shifts have been observed in other grassy ecosystems (Fidelis et al., 2014; Bombo et al., 2022a, 2022b), reinforcing the idea that fire regimes play a central role in shaping the belowground traits associated with post‐fire recovery dynamics of open ecosystems.

Contrary to what is observed in tropical savannas (Le Stradic et al., 2021), we recorded a decrease in the root biomass with increasing fire frequency, while belowground organ biomass was not affected. Campos rupestres are dominated by grass and sedge species that produce substantial root biomass at the soil surface in shallow soils (Díaz et al., 2016; Brum et al., 2017). Recurrent fire may damage these superficial roots, while the limited nutrient content of campos rupestres does not support rapid root production post fire. Conversely, fire exclusion benefits grass species, as shown by the increase in graminoid fleshy rhizomes under these conditions. The dense root systems of grasses accumulate at the soil surface, and root biomass increases in the absence of fire. Species with fleshy rhizomes, such as some grass species, lack substantial reserves and depend on fine roots to capture resources, whereas species with organs like xylopodia do not rely heavily on root production to acquire resources (Pausas et al., 2018).

Although aboveground biomass is known to be affected by fire frequency, with recurrent fires reducing the total amount of aboveground biomass (Rodrigues et al., 2021), the belowground‐to‐aboveground ratio, both organ‐to‐shoot and root‐to‐shoot, presented values that were consistently greater than one in our study. These uniformly high belowground‐to‐aboveground ratios highlight the predominance of belowground biomass allocation in campos rupestres, regardless of fire frequency. Such a pattern can be associated with a resource‐conservative strategy, characterized by biomass accumulation in specialized belowground organs, in addition to the roots, and is a well‐documented adaptation in savannas and other open grassy ecosystems (Fidelis et al., 2013; Klimešová et al., 2018, 2021; Le Stradic et al., 2021; Ottaviani et al., 2024). In these systems, belowground plant organs constitute a substantial portion of total plant biomass, which results in higher belowground‐to‐shoot ratios (Ottaviani et al., 2024). These patterns indicate that the soil serves a major reservoir of organic biomass in old‐growth grasslands.

## CONCLUSIONS

Alterations in fire regimes, such as increasing fire frequency, can lead to drastic changes in the belowground compartment of old‐growth grasslands like the campos rupestres. An increase in fire frequency leads to a significant reduction of the bud bank size and bud‐bearing organ density, which may hamper regeneration and threaten the resilience of the campos rupestres. Our findings indicate that forbs in the campos rupestres can be particularly sensitive to an increase in fire frequency, as their belowground bud‐bearing organs, i.e., non‐graminoid fleshy rhizomes, are especially vulnerable to high fire frequency. Forbs are the most taxonomically and functionally diverse group in tropical old‐growth grasslands (Siebert et al., 2024), and a key source of diversity and endemism in the campos rupestres (Silveira et al., 2016). However, they remain underrepresented in most restoration projects worldwide due to limited understanding of their dynamics (Nerlekar et al., 2024), particularly their belowground components. Fire management needs to be planned for the conservation and restoration of the campos rupestres to ensure plant diversity and community resilience.

## AUTHOR CONTRIBUTIONS

A.B.B., A.F., and S.L.S. conceived and designed the research. A.B.B. and S.L.S. collected and analyzed the data and drafted the manuscript. A.F. provided critical revisions. All authors contributed significantly to the drafts and approved the final version for publication.

## Supporting information


**Appendix S1.** Supporting figure and tables.
**Figure S1.** Location of the three selected sites that differ fire frequency in the campos rupestres (map) and image of each site.
**Table S1.** Information about the three sampling sites in campos rupestres.
**Table S2.** Bud bank density, total and by growth form, number of live belowground bud‐bearing organs, and number of each belowground organ type in campos rupestres under different fire frequencies.
**Table S3.** Results of PERMANOVA and pairwise comparison tests of the differences in belowground organ composition between fire frequencies based on Bray–Curtis dissimilarities and 9.999 permutations.

## Data Availability

The data supporting this study are available in Zenodo (https://doi.org/10.5281/zenodo.16780341).

## References

[ajb270108-bib-0001] Alvarado, S. T. , T. Fornazari , A. Cóstola , L. P. C. Morellato , and T. S. F. Silva . 2017. Drivers of fire occurrence in a mountainous Brazilian Cerrado savanna: tracking long‐term fire regimes using remote sensing. Ecological Indicators 78: 270–281.

[ajb270108-bib-0002] Alvarado, S. T. , T. S. F. Silva , and S. Archibald . 2018. Management impacts on fire occurrence: a comparison of fire regimes of African and South American tropical savannas in different protected areas. Journal of Environmental Management 218: 79–87. 29665489 10.1016/j.jenvman.2018.04.004

[ajb270108-bib-0003] Andrino, C. O. , F. N. Costa , and P. T. Sano . 2015. The genus *Paepalanthus* Mart. (Eriocaulaceae) at Biribiri State Park, Diamantina, Minas Gerais, Brazil. Rodriguesia 66: 393–419.

[ajb270108-bib-0004] Appezzato‐da‐Glória, B. , and G. Cury . 2011. Morpho‐anatomical features of underground systems in six Asteraceae species from the Brazilian Cerrado. Anais da Academia Brasileira de Ciências 83: 981–992. 21779656 10.1590/s0001-37652011005000018

[ajb270108-bib-0005] Araújo, T. , and A. A. Conceição . 2021. High functional redundancy drives vegetation recovery in campo rupestre affected by wildfires. Flora: Morphology, Distribution, Functional Ecology of Plants 281: 151866.

[ajb270108-bib-0006] Bellingham, P. J. , and A. D. Sparrow . 2000. Resprouting as a life history strategy in woody plant communities. Oikos 89: 409–416.

[ajb270108-bib-0007] Bombo, A. B. , B. Appezzato‐da‐Glória , and A. Fidelis . 2022a. Fire exclusion changes belowground bud bank and bud‐bearing organ composition jeopardizing open savanna resilience. Oecologia 199: 153–164. 35471620 10.1007/s00442-022-05172-1

[ajb270108-bib-0008] Bombo, A. B. , B. Appezzato‐da‐Glória , R. Martins , and A. Fidelis . 2024. Belowground organs and bud bank: insights on morphoanatomical functional traits related to fire. Folia Geobotanica 58: 259–273.

[ajb270108-bib-0009] Bombo, A. B. , F. Siebert , and A. Fidelis . 2022b. Fire and herbivory shape belowground bud banks in a semi‐arid African savanna. African Journal of Range & Forage Science 39: 16–26.

[ajb270108-bib-0010] Brooks, M. E. , K. Kristensen , K. J. van Benthem , A. Magnusson , C. W. Berg , A. Nielsen , H. J. Skaug , et al. 2017. glmmTMB balances speed and flexibility among packages for zero‐inflated generalized linear mixed modeling. R Journal 9: 378–400.

[ajb270108-bib-0011] Brum, M. , G. S. Teodoro , A. Abrahão , and R. S. Oliveira . 2017. Coordination of rooting depth and leaf hydraulic traits defines drought‐related strategies in the campos rupestres, a tropical montane biodiversity hotspot. Plant and Soil 420: 467–480.

[ajb270108-bib-0012] Buisson, E. , S. Archibald , A. Fidelis , and K. N. Suding . 2022. Ancient grasslands guide ambitious goals in grassland restoration. Science 377: 594–598. 35926035 10.1126/science.abo4605

[ajb270108-bib-0013] Buisson, E. , S. Le Stradic , F. A. O. Silveira , G. Durigan , G. E. Overbeck , A. Fidelis , G. W. Fernandes , et al. 2019. Resilience and restoration of tropical and subtropical grasslands, savannas, and grassy woodlands. Biological Reviews 94: 590–609. 30251329 10.1111/brv.12470

[ajb270108-bib-0014] Chiminazzo, M. , A. Bertolosi Bombo , T. Charles‐Dominique , and A. Fidelis . 2023. Bark production of generalist and specialist species across savannas and forests in the Cerrado. Annals of Botany 131: 613‐621. 36651635 10.1093/aob/mcad014 PMC10147323

[ajb270108-bib-0015] Cozin, B. B. , T. C. Ferreira , L. F. Daibes , I. F. de Carvalho , B. S. dos Santos , R. P. de Souza , L. S. de Camargos , and A. R. Martins . 2024. Unveiling the hidden reserves: allocation strategies associated with underground organs of Cerrado legumes in fire‐prone savannas. Functional Plant Biology 51: FP24104. 39163498 10.1071/FP24104

[ajb270108-bib-0016] Díaz, S. , J. Kattge , J. H. C. Cornelissen , I. J. Wright , S. Lavorel , S. Dray , B. Reu , et al. 2016. The global spectrum of plant form and function. Nature 529: 167–171. 26700811 10.1038/nature16489

[ajb270108-bib-0017] Dutilh, J. H. A. 2005. Ornamental bulbous plants of Brazil. Acta Horticulturae 683: 37–42.

[ajb270108-bib-0018] Eiten, G. 1982. Brazilian “savannas”. *In* B. J. Hultley and B. H. Walker [eds.], Ecology of tropical savannas, 25–47. Springer‐Verlag, Berlin, Germany.

[ajb270108-bib-0019] Ferraro, A. , A. Fidelis , G. S. da Silva , A. R. Martins , S. M. D. S. Piedade , and B. Appezzato‐da‐Glória . 2021. Long‐term *Pinus* plantations reduce the bud bank in Cerrado areas, Applied Vegetation Science 24: avsc.12537.

[ajb270108-bib-0020] Fidelis, A. , B. Appezzato‐da‐Glória , V. D. Pillar , and J. Pfadenhauer . 2014. Does disturbance affect bud bank size and belowground structures diversity in Brazilian subtropical grasslands? Flora 209: 110–116.

[ajb270108-bib-0021] Fidelis, A. , M. F. di S. Lyra , and V. R. Pivello . 2013. Above‐ and below‐ground biomass and carbon dynamics in Brazilian Cerrado wet grasslands. Journal of Vegetation Science 24: 356–364.

[ajb270108-bib-0022] Figueira, J. E. C. , K. T. Ribeiro , M. C. Ribeiro , C. M. Jacobi , H. França , A. C. de Oliveira Neves , A. A. Conceição , et al. 2016. Fire in rupestrian grasslands: plant response and management. *In* G. W. Fernandes [ed.], Ecology and conservation of mountaintop grasslands in Brazil, 415–448. Springer International, Cham, Switzerland.

[ajb270108-bib-0023] Figueira, J. E. C. , and M. C. L. Del Sarto . 2007. Clonal growth and dispersal potential of *Leiothrix flagellaris* Ruhland (Eriocaulaceae) in the rocky grasslands of southeastern Brazil. Revista Brasileira de Botânica 4: 679–686.

[ajb270108-bib-0024] Fox, J. , and S. Weisberg . 2019. An R companion to applied regression, 3rd ed. Sage, Thousand Oaks, CA, USA.

[ajb270108-bib-0025] Giulietti, A. M. , N. L. Menezes , J. R. Pirani , M. Meguro , and M. G. L. Wanderley . 1987. Flora da Serra do Cipó, Minas Gerais: caracterização e lista das espécies. Boletim de Botânica 9: 1.

[ajb270108-bib-0026] Hartig, F. 2020. DHARMa: residual diagnostics for hierarchical (multi‐level/mixed) regression models. R package version 0.4.6. Website: https://CRAN.R-project.org/package=DHARMa [accessed 5 August 2025].

[ajb270108-bib-0027] Jackman, S. 2020. pscl: Classes and methods for R developed in the Political Science Computational Laboratory. University of Sydney. Sydney, New South Wales, Australia. R package version 1.5.5. Website: https://github.com/atahk/pscl/ [accessed 05 August 2025].

[ajb270108-bib-0028] Joaquim, E. de O. , T. M. Silva , R. de C. L. Figueiredo‐Ribeiro , M. G. de Moraes , and M. A. M. de . Carvalho . 2018. Diversity of reserve carbohydrates in herbaceous species from Brazilian campo rupestre reveals similar functional traits to endure environmental stresses. Flora 238: 201–209.

[ajb270108-bib-0029] José, R. , and V. Alves . 1994. Morphological age determination and longevity in some *Vellozia* populations in Brazil. Folia Geobotanica & Phytotaxonomica 29: 55‐59.

[ajb270108-bib-0030] Klimešová, J. , J. Martínková , and G. Ottaviani . 2018. Belowground plant functional ecology: towards an integrated perspective. Functional Ecology 32: 2115–2126.

[ajb270108-bib-0031] Klimešová, J. , J. Martínková , J. G. Pausas , M. G. de Moraes , T. Herben , F. H. Yu , J. Puntieri , et al. 2019. Handbook of standardized protocols for collecting plant modularity traits. Perspectives in Plant Ecology, Evolution and Systematics 40: 125485.

[ajb270108-bib-0032] Klimešová, J. , O. Mudrák , J. Martínková , A. Lisner , J. Lepš , A. L. Filartiga , and G. Ottaviani . 2021. Are belowground clonal traits good predictors of ecosystem functioning in temperate grasslands? Functional Ecology 35: 787‐795.

[ajb270108-bib-0033] Lüdecke, D. 2024. sjPlot: data visualization for statistics in social science. R package version 2.8.16. Website: https://CRAN.R-project.org/package=sjPlot [accessed 5 August 2025].

[ajb270108-bib-0034] Lüdecke, D. , M. Ben‐Shachar , I. Patil , P. Waggoner , and D. Makowski . 2021. Performance: an R package for assessment, comparison and testing of statistical models. Journal of Open Source Software 6: 3139.

[ajb270108-bib-0035] Lusa, M. A. G. , B. A. Appezzato‐da‐Glória , B. C. Loeuille , G. D. Bartoli , and D. D. Ciccarelli . 2014. Functional groups in Lychnophorinae (Asteraceae: Vernonieae) based on morphological and anatomical traits. Australian Journal of Botany 62: 150–163.

[ajb270108-bib-0036] Makowski, D. , D. Lüdecke , I. Patil , R. Thériault , M. Ben‐Shachar , and B. Wiernik . 2023. Automated results reporting as a practical tool to improve reproducibility and methodological best practices adoption. CRAN. Website: https://easystats.github.io/report/ [accessed 5 August 2025].

[ajb270108-bib-0037] Moraes, M. , M. A. M. de Carvalho , A. C. Franco , C. J. Pollock , and R. de C. L. Figueiredo‐Ribeiro . 2016. Fire and drought: soluble carbohydrate storage and survival mechanisms in herbaceous plants from the cerrado. BioScience 66: 107–117.

[ajb270108-bib-0038] Nerlekar, A. N. , L. L. Sullivan , and L. A. Brudvig . 2024. Grassland restorations must better foster forbs to facilitate high biodiversity. Restoration Ecology 32: e14214.

[ajb270108-bib-0039] Nerlekar, A. N. , and J. W. Veldman . 2020. High plant diversity and slow assembly of old‐growth grasslands. Proceedings of the National Academy of Sciences, USA 117: 18550–18556. 10.1073/pnas.1922266117 PMC7414179 32675246

[ajb270108-bib-0040] Neves, S. P. S. , and A. A. Conceição . 2010. Campo rupestre recém‐queimado na Chapada Diamantina, Bahia, Brasil: plantas de rebrota e sementes, com espécies endêmicas na rocha. Acta Botanica Brasilica 24: 697–707.

[ajb270108-bib-0041] Oliveira, R. S. , A. Abrahão , C. Pereira , G. S. Teodoro , M. Brum , S. Alcantara , and H. Lambers . 2016. Ecophysiology of Campos rupestres plants. *In* G. Fernandes . [ed.], Ecology and Conservation of Mountaintop Grasslands in Brazil, 227–272. Springer, Cham.

[ajb270108-bib-0042] Oliveira, R. S. , H. C. Galvão , M. C. R. de Campos , C. B. Eller , S. J. Pearse , and H. Lambers . 2015. Mineral nutrition of campos rupestres plant species on contrasting nutrient‐impoverished soil types. New Phytologist 205: 1183–1194. 25425486 10.1111/nph.13175

[ajb270108-bib-0043] Oliveras, I. , S. T. Meirelles , V. L. Hirakuri , C. R. Freitas , H. S. Miranda , and V. R. Pivello . 2012. Effects of fire regimes on herbaceous biomass and nutrient dynamics in the Brazilian savanna. International Journal of Wildland Fire 22: 368–380.

[ajb270108-bib-0044] Oksanen, J. , G. L. Simpson , F. G. Blanchet , R. Kindt , P. Legendre , P. R. Minchin , R. B. O'Hara , et al. 2025. vegan: Community ecology package. R package version 2.7‐1. Website: https://CRAN.R-project.org/package=vegan [accessed 5 August 2025].

[ajb270108-bib-0045] Ott, J. P. , J. Klimešová , and D. C. Hartnett . 2019. The ecology and significance of below‐ground bud banks in plants. Annals of Botany 123: 1099–1118. 31167028 10.1093/aob/mcz051 PMC6612937

[ajb270108-bib-0046] Ottaviani, G. , J. Klimešová , T. Charles‐Dominique , M. Millan , T. Harris , and F. A. O. Silveira . 2024. The underestimated global importance of plant belowground coarse organs in open biomes for ecosystem functioning and conservation. Perspectives in Ecology and Conservation 22: 118–121.

[ajb270108-bib-0047] Pausas, J. G. , B. B. Lamont , S. Paula , B. Appezzato‐da‐Glória , and A. Fidelis . 2018. Unearthing belowground bud banks in fire‐prone ecosystems. New Phytologist 217: 1435–1448. 29334401 10.1111/nph.14982

[ajb270108-bib-0048] Pérez‐Harguindeguy, N. , S. Díaz , E. Garnier , S. Lavorel , H. Poorter , P. Jaureguiberry , M. S. Bret‐Harte , et al. 2013. New handbook for standardised measurement of plant functional traits worldwide. Australian Journal of Botany 61: 167–234.

[ajb270108-bib-0049] Pivello, V. R. , I. Vieira , A. V. Christianini , D. B. Ribeiro , L. da Silva Menezes , C. N. Berlinck , F. P. L. Melo , et al. 2021. Understanding Brazil's catastrophic fires: causes, consequences and policy needed to prevent future tragedies. Perspectives in Ecology and Conservation 19: 233–255.

[ajb270108-bib-0050] R Core Team . 2019. R: a language and environment for statistical computing. R Foundation for Statistical Computing, Vienna, Austria. Website: https://www.R-project.org/ [accessed 5 August 2025].

[ajb270108-bib-0051] Rodrigues, C. A. , H. L. Zirondi , and A. Fidelis . 2021. Fire frequency affects fire behavior in open savannas of the Cerrado. Forest Ecology and Management 482: 118850.

[ajb270108-bib-0052] Senande‐Rivera, M. , D. Insua‐Costa , and G. Miguez‐Macho . 2022. Spatial and temporal expansion of global wildland fire activity in response to climate change. Nature Communications 13: 1208. 10.1038/s41467-022-28835-2 PMC8904637 35260561

[ajb270108-bib-0053] Siebert, F. , M. Te Beest , R. Fynn , J. Klimešová , C. Morris , S. Nkuna , S. Siebert , and A. Fidelis . 2024. Past, present, and future of forbs in old‐growth tropical and subtropical grasslands. Annual Review of Ecology 22: 56.

[ajb270108-bib-0054] Silva, G. S. , A. Ferraro , C. Lima de Aguiar , and B. Appezzato‐da‐Glória . 2021. Resprouting strategies of three native shrub cerrado species from a morphoanatomical and chemical perspective. Australian Journal of Botany 69: 527–542.

[ajb270108-bib-0055] Silva, K. C. , M. Brum , R. S. Oliveira , B. V. Barbosa , V. Negrão‐Rodrigues , G. S. Teodoro , and E. L. Rowland . 2023. High resilience of campos rupestres plants to the interaction of drought and fire. Plant Biology X: 1. 10.1111/plb.13596 38059684

[ajb270108-bib-0056] Silveira, F. A. O. , D. Negreiros , N. P. U. Barbosa , E. Buisson , F. F. Carmo , D. W. Carstensen , A. A. Conceição , et al. 2016. Ecology and evolution of plant diversity in the endangered campo rupestre: a neglected conservation priority. Plant and Soil 403: 129–152.

[ajb270108-bib-0057] Simon, M. F. , and T. Pennington . 2012. Evidence for adaptation to fire regimes in the tropical savannas of the Brazilian cerrado. International Journal of Plant Sciences 173: 711–723.

[ajb270108-bib-0058] Le Stradic, S. , E. Buisson , G. W. Fernandes , and L. P. C. Morellato . 2018a. Reproductive phenology of two co‐occurring Neotropical mountain grasslands. Journal of Vegetation Science 29: 15–24.

[ajb270108-bib-0059] Le Stradic, S. , G. W. Fernandes , and E. Buisson . 2018b. No recovery of campo rupestre grasslands after gravel extraction: implications for conservation and restoration. Restoration Ecology 26: S151–S159.

[ajb270108-bib-0060] Le Stradic, S. , P. Hernandez , G. W. Fernandes , and E. Buisson . 2018c. Regeneration after fire in campo rupestre: short‐ and long‐term vegetation dynamics. Flora 238: 191–200.

[ajb270108-bib-0061] Le Stradic, S. , C. Roumet , G. Durigan , L. Cancian , and A. Fidelis . 2021. Variation in biomass allocation and root functional parameters in response to fire history in Brazilian savannas. Journal of Ecology 109: 4143–4157.

[ajb270108-bib-0062] Le Stradic, S. , F. A. O. Silveira , E. Buisson , K. Cazelles , V. Carvalho , and G. W. Fernandes . 2015. Diversity of germination strategies and seed dormancy in herbaceous species of campo rupestre grasslands. Austral Ecology 40: 537–546.

[ajb270108-bib-0063] Teodoro, G. S. , P. de Britto Costa , M. Brum , C. Signori‐Müller , S. Alcantara , T. E. Dawson , A. G. West , et al. 2021. Desiccation tolerance implies costs to productivity but allows survival under extreme drought conditions in Velloziaceae species in campos rupestres. Environmental and Experimental Botany 189: 104556.

[ajb270108-bib-0064] Veldman, J. W. , E. Buisson , G. Durigan , G. W. Fernandes , S. Le Stradic , G. Mahy , D. Negreiros , et al. 2015. Toward an old‐growth concept for grasslands, savannas, and woodlands. Frontiers in Ecology and the Environment 13: 154–162.

[ajb270108-bib-0065] Vesk, P. A. , and M. Westoby . 2004. Sprouting ability across diverse disturbances and vegetation types worldwide. Journal of Ecology 92: 310–320.

[ajb270108-bib-0066] Wanderley, M. das G. L. 2010. Cinco novas espécies de *Xyris* (Xyridaceae) da Serra do Cipó, Minas Gerais, Brasil. Rodriguésia 1: 83–94.

[ajb270108-bib-0067] Wickham, H. 2016. ggplot2: elegant graphics for data analysis, 2nd ed. Springer‐Verlag, NY, NY, USA.

[ajb270108-bib-0068] Wickham, H. , M. Averick , J. Bryan , W. Chang , L. D'Agostino McGowan , R. François , et al. 2019. Welcome to the Tidyverse. Journal of Open Source Software 4: 1686.

[ajb270108-bib-0069] Zirondi, H. L. , H. P. José , L. F. Daibes , and A. Fidelis . 2019a. Heat and smoke affect the germination of flammable resprouters: *Vellozia* species in the Cerrado. Folia Geobotanica 54: 65–72.

[ajb270108-bib-0070] Zirondi, H. L. , F. A. O. Silveira , and A. Fidelis . 2019b. Fire effects on seed germination: heat shock and smoke on permeable vs impermeable seed coats. Flora: Morphology, Distribution, Functional Ecology of Plants 253: 98–106.

[ajb270108-bib-0071] Zuur, A. F. , E. N. Ieno , N. J. Walker , A. A. Saveliev , and G. M. Smith . 2009. Mixed effects models and extensions in ecology with R. Springer, NY, NY, USA.

