# Supplementary material for: Modifications in fire frequency impact belowground plant components in old‐growth grasslands, posing risks to their resilience
Source: Am J Bot. 2025 Oct 10;112(10):e70108. doi: 10.1002/ajb2.70108 (PMC12572682; doi:10.1002/ajb2.70108)
Supplement: Supplementary file 1 — Appendix S1. Supporting figure and tables. Figure S1. Location of the three selected sites that differ fire frequency in the campos rupestres (map) and image of each site. Table S1. Information about the three sampling sites in campos rupestres. Table S2. Bud bank density, total and by growth form, number of live belowground bud‐bearing organs, and number of each belowground organ type in campos rupestres under different fire frequencies. Table S3. Results of PERMANOVA and pairwise comparison tests of the differences in belowground organ composition between fire frequencies based on Bray–Curtis dissimilarities and 9.999 permutations. [file AJB2-112-e70108-s001.docx]

**Bombo et al. – American Journal of Botany 2025 – Appendix S1**

**Appendix S1.** Supporting figure and tables.


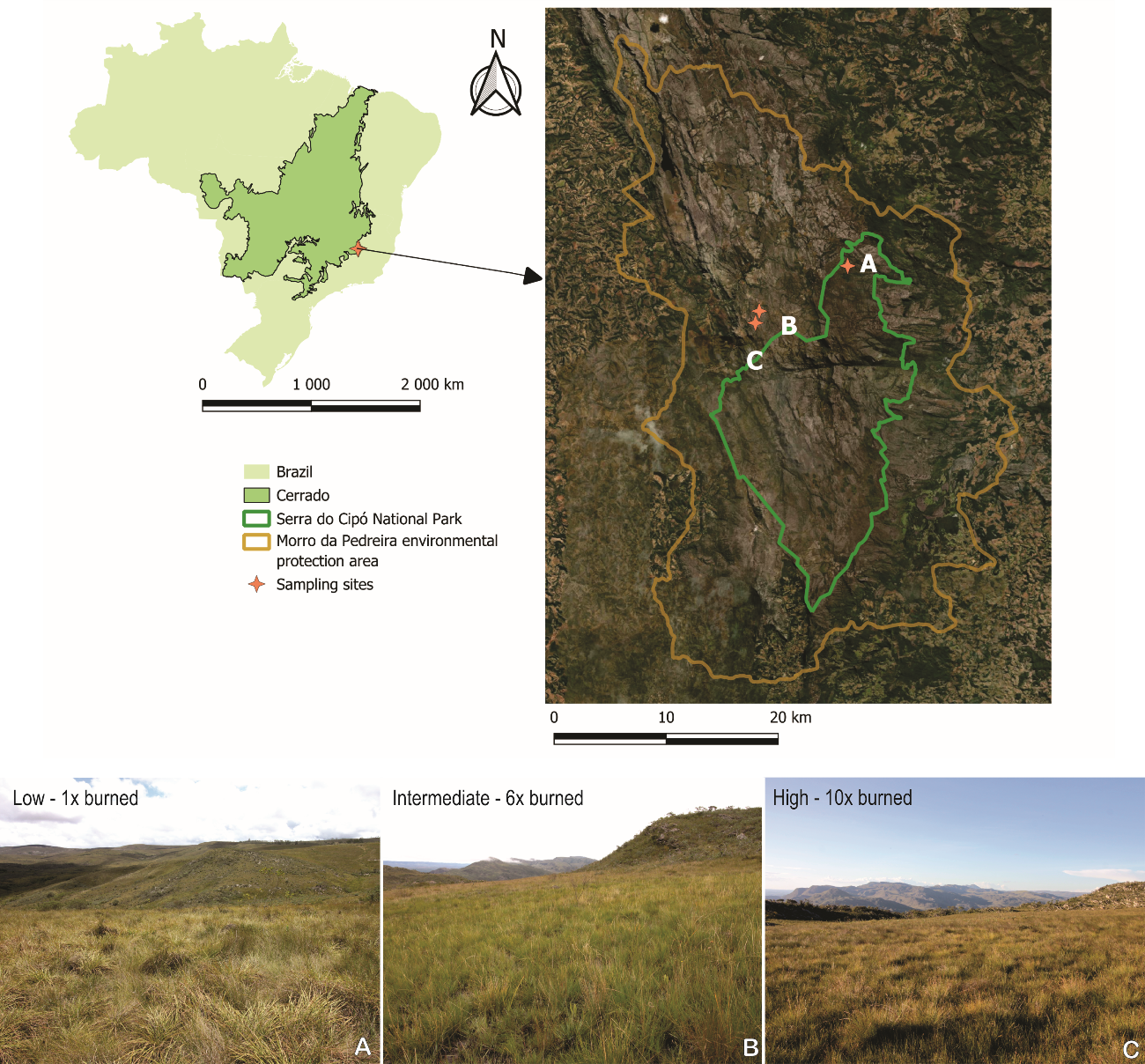


Figure S1. Geographic distribution of the three selected sites that differ in fire frequency in the campos rupestres (map) and photograph of each site (A–C). A–C on the map correspond to images A–C. The number of fires between 1984 and 2018 is indicated at the top left of each image.

**Table S1** Information about the three sampling sites in campos rupestres, including geographical coordinates (GPS), fire frequency, number of fires (between 1984 and 2018), time since the last fire, and years of fire events.

| Site | GPS | Fire frequency | Number of fires (1984–2018) | Time since last fire (years) | Years of fires |
| --- | --- | --- | --- | --- | --- |
| A | S19.24786° W43.51361° | Low | 1 | 19 | 1999 |
| B | S19.28638° W43.58838° | Intermediate | 6 | 4 | 1984, 1986,1989, 1993, 1997, 2014 |
| C | S19.29616° W43.59180° | High | 10 | 4 | 1984, 1986, 1988, 1993, 1996, 1999, 2006, 2007, 2011, 2014 |

**Table S2** Bud bank density, total and by type, number of live belowground bud-bearing organs, and number of each belowground organ type in sites with different fire frequencies in the campos rupestres. Generalized linear mixed models were used to analyze differences in the belowground bud bank and organ types in relation to the number of fires (1984–2018). Incidence rate ratios for the intercept and treatment and the *P*-value are given for each model. The intercept represents the baseline incidence rate; fire frequency effect reflects the relative change in the incidence associated with the fire frequency (**P* < 0.05; ***P* < 0.01; ****P* < 0.001; n.s., nonsignificant). Different letters represent treatment differences identified by Tukey’s HSD test.

| No. fires | Total bud bank (no. buds m^–2^) | Graminoids bud bank (no. buds m^–2^) | Non-graminoids bud bank each linear mixed-models (no. buds m^–2^) | Density of live organs density (units m^–2^) | No. rhizomes (graminoids) | No. rhizomes (non-graminoids) | No. bulbs | No. root crowns | No. other structures | No. xylopodia | No. woody rhizomes |
| --- | --- | --- | --- | --- | --- | --- | --- | --- | --- | --- | --- |
| 1 | 3435 ± 370 a | 2900 ± 349 a | 535 ± 121 a | 1037 ± 95 a | 740 ± 77 a | 279 ± 63 a | 6 ± 4 a | 2 ± 1 a | 9 ± 4 a | 1 ± 1 a | 0 ± 0 a |
| 6 | 2486 ± 302 ab | 1928 ± 193 b | 557 ± 181 a | 696 ± 65 b | 544 ± 50 ab | 131 ± 23 a | 2 ± 1 a | 6 ± 3 a | 8 ± 4 a | 1 ± 1 a | 4 ± 4 ab |
| 10 | 2337 ± 252 b | 2168 ± 263 ab | 169 ± 55 b | 589 ± 53 b | 531 ± 49 b | 35 ± 12 b | 0 ± 0 a | 1 ± 1 a | 5 ± 2 a | 6 ± 5 a | 10 ± 5 b |
|  | Model estimates | | | | | | | | | | |
| (Intercept) | 3258.65 *** | 2602.21 *** | 566.78 *** | 1035.27 *** | 710.20 *** | 324.85 *** | 36.11 *** | 6.40 *** | 15.9 *** | 9.46 *** | 80.95 *** |
| Fire frequency | 0.96 * | 0.97 n.s. | 0.86 ** | 0.94 *** | 0.96 * | 0.76 *** | 0.78 *** | 1.05 n.s. | 0.96 n.s. | 1.05 n.s. | 0.89 * |

**Table S3.** Results of PERMANOVA and pairwise comparison tests of the differences in belowground organ composition between fire frequencies (1, 6, 10 fire events 1984–2018) based on Bray–Curtis dissimilarities and 9.999 permutations. **P* < 0.05; ***P* < 0.01; ****P* < 0.001; n.s., nonsignificant

| **Test** | **Comparison** | **df** | **Sum of Squares** | ***R*^2^** | ***F*** | ***P*** | **Significance** |
| --- | --- | --- | --- | --- | --- | --- | --- |
| PERMANOVA | Overall | 1 | 0.20191 | 0.30522 | 12.3 | 0.0011 | ** |
| Pairwise test | 10 vs 6 | 1 | 0.09781 | 0.29474 | 7.52 | 0.021 | * |
|  | 10 vs 1 | 1 | 0.20868 | 0.44145 | 14.23 | 0.003 | ** |
|  | 6 vs 1 | 1 | 0.01724 | 0.04198 | 0.79 | 1.000 | n.s. |
